# Dynamics of RAS Mutations in Liquid Biopsies in Metastatic Colorectal Cancer Patients—Case Series and Literature Review

**DOI:** 10.3390/jpm14070750

**Published:** 2024-07-15

**Authors:** Ionut Popescu, Vlad M. Croitoru, Irina M. Croitoru-Cazacu, Ana-Maria Dudau, Vlad Herlea, Simona Olimpia Dima, Adina Emilia Croitoru

**Affiliations:** 1 Faculty of Medicine, Titu Maiorescu University, 031593 Bucharest, Romania; r.ionut.popescu@gmail.com (I.P.); vlad.m.croitoru@gmail.com (V.M.C.);; 2 Department of Oncology, Fundeni Clinical Institute, 022328 Bucharest, Romania; 3 Faculty of Medicine, Carol Davila University of Medicine and Pharmacy, 020021 Bucharest, Romaniadima.simona@gmail.com (S.O.D.); 4 Department of Pathology, Fundeni Clinical Institute, 022328 Bucharest, Romania; 5 Center of Excellence in Translational Medicine, Fundeni Clinical Institute, 022328 Bucharest, Romania

**Keywords:** metastatic colorectal cancer, RAS wild-type, liquid biopsy, KRAS, ctDNA

## Abstract

Liquid biopsies can accurately identify molecular alterations in patients with colorectal cancer with high concordance with tissue analysis and shorter turnaround times. Circulating tumor (ct) DNA analysis can be used for diagnosing and monitoring tumor evolution in patients with metastatic colorectal cancer who are treated with EGFR inhibitors. In this article, we reported three clinical cases to illustrate the relevance of RAS mutations identified in ctDNA samples of patients with wild-type metastatic colorectal cancer who received an EGFR inhibitor plus chemotherapy as first-line treatment. The identification of RAS mutations in these patients is one of the most frequently identified mechanisms of acquired resistance. However, detecting a KRAS mutation via liquid biopsy can be caused by inter-tumor heterogeneity or it can be a false positive due to clonal hematopoiesis. More research is needed to determine whether ctDNA monitoring may help guide therapy options in metastatic colorectal cancer patients. We performed a literature review to assess the technologies that are used for analysis of RAS mutations on ctDNA, the degree of agreement between tissue and plasma and the importance of tissue/plasma discordant cases.

## 1. Introduction

Colorectal cancer (CRC) is the third most common and the second most deadly cancer worldwide, representing 10.7% of new cases and 9.5% of cancer-related deaths [1]. The treatment of CRC is mainly driven by the stage of the cancer, the patient’s clinical condition and certain molecular characteristics of the tumor. In patients with metastatic disease, the preferred therapeutic option is systemic therapy, which may include chemotherapy, targeted agents or immunotherapy. In the era of personalized medicine, it is essential to identify biomarkers that will inform the choice of appropriate therapeutic agents.

This process entails the examination of genomic alterations, including KRAS/NRAS/BRAF mutations, and microsatellite instability (MSI)/mismatch repair genes (MMR). Consequently, patients exhibiting wild-type KRAS, NRAS and BRAFV600E genotypes are likely to benefit from the administration of chemotherapy doublets, including FOLFOX (5-Fluouracil, folinic acid and oxaliplatin), CAPOX (capecitabine and oxaliplatin) and FOLFIRI (5-Fluouracil, folinic acid and irinotecan), in conjunction with EGFR-inhibitors such as Cetuximab or Panitumumab [2]. Anti-EGFR monoclonal antibody (moAb) Cetuximab comprises a chimeric immunoglobulin G1 (IgG1), which, upon binding to the EGFR receptor, initiates the internalization and degradation of the receptor, thus disrupting the downstream pathway. Cetuximab is also capable of eliciting immune functions such as antibody-dependent cell-mediated cytotoxicity (ADCC), which serves as an anti-tumor effect [3]. Nevertheless, Panitumumab, a humanized IgG2 monoclonal antibody with a similar mechanism of action, is unable to elicit an ADCC effect, but in return it makes it less likely to determine secondary reactions [4].

The detection of molecular alterations has enabled personalized treatment in oncological diseases. Mutations in RAS and BRAF genes are widely recognized as the primary drivers of resistance to anti-EGFR agents in metastatic colorectal cancer (mCRC). Testing for these mutations is essential as they serve as predictive biomarkers for both primary and acquired resistance [5]. Primary resistance refers to the presence of activating mutations in these genes prior to treatment initiation, while the selection of pre-existing mutant clones or de novo acquisition of mutations under the influence of anti-EGFR treatment is known as acquired resistance [6]. The identification of molecular mechanisms behind acquired resistance to cetuximab or panitumumab in mCRC is crucial for predicting the failure of anti-EGFR antibodies and developing new treatment approaches.

Cancers are defined by the simultaneous presence of various genetic changes, resulting in the occurrence of spatial and temporal heterogeneity [6,7]. While tissue biopsy is the gold standard and preferred method for cancer diagnosis, it may not fully capture the spatial and temporal heterogeneity of the tumor. Tissue biopsies are invasive procedures, associated with risks of complications, low patient compliance and substantial procedural expenses [6].

When compared to repeated tumor tissue biopsies, the use of circulating tumor (ct) DNA for genotyping the tumor (liquid biopsy) is less invasive and does not require a lesion accessible to biopsy. Furthermore, liquid biopsy is able to capture the tumor heterogeneity that appears during treatment before radiological progression [8]. Thus, liquid biopsy could be a valuable method for assessing the presence and heterogeneity of the tumor, as well as monitoring cancer treatment.

Studies have demonstrated that ctDNA analysis can accurately identify molecular alterations in patients with CRC with high concordance with tissue analysis and shorter turnaround times [9,10,11,12,13,14]. Recent studies have demonstrated the utility of ctDNA analysis for diagnosing and monitoring tumor evolution in patients with mCRC who are treated with EGFR inhibitors. In this patient population, RAS/BRAF-acquired mutations were detected at the time of disease progression [15,16,17].

The case series reported in this article aim to illustrate the clinical relevance of RAS mutations identified in ctDNA samples of patients with mCRC with a baseline RAS wild-type tumor tissue and who received an anti-EGFR monoclonal antibody plus chemotherapy as first-line treatment. Furthermore, we performed a literature review to assess the technologies that are used for analysis of RAS mutations on ctDNA, the degree of agreement between tissue and plasma and the importance of tissue/plasma discordant cases.

## 2. Methods

### 2.1. Case Series

**Figure 1:**
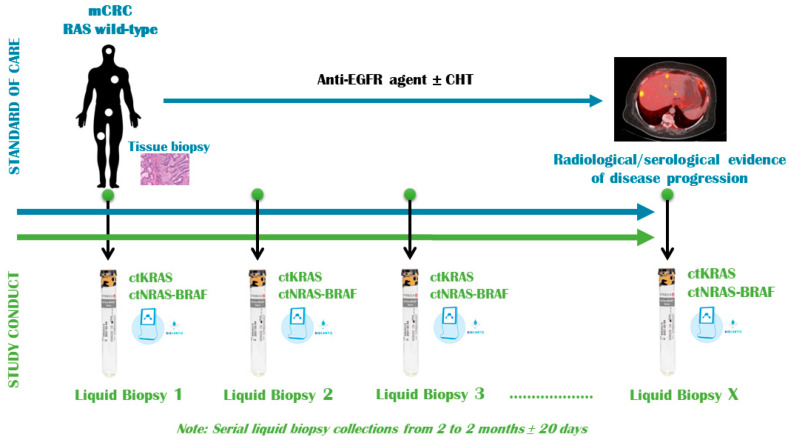
Ongoing prospective study design. mCRC, metastatic colorectal cancer; CHT, chemotherapy.

The patients reported in this article were enrolled in an ongoing prospective study conducted at our institution (Fundeni Clinical Institute) that aims to assess the mutational status of KRAS, NRAS and BRAF on liquid biopsy at predefined intervals using the Idylla© Biocartis (Mechelen, Belgium)system in patients with mCRC (Figure 1). Mutational analysis was performed using the molecular biology equipment real time (rt) PCR Idylla (Supplementary Material) and the following related tests:

ctKRAS test—it can detect 21 clinically relevant mutations (plasma/liquid biopsy)—Exon 2 (codon 12, 13), Exon 3 (codon 59, 61) Exon 4 (codon 117, 146)

ctNRAS-BRAF test—it can detect 23 clinically relevant mutations (plasma/liquid biopsy)—NRAS (18 mutations)—Exon 2 (codon 12, 13), Exon 3 (codon 59, 61), Exon 4 (codon 117, 146); BRAF (5 mutations)—Exon 15, codon 600 (BRAF V600E, BRAF V600D, BRAF V600K, BRAF V600 R)

The performance of the rtPCR Idylla© Biocartis platform as a method for determining these mutations has been highlighted by numerous studies [14,18].

### 2.2. Literature Search Strategy

A literature search was conducted in PubMed and Web of Science databases in February 2024, using the following key terms: colorectal cancer, colon cancer, metastatic colorectal cancer/mCRC, RAS, KRAS, NRAS, liquid biopsy, circulating tumor DNA, cell-free DNA. Relevant medical subject headings terms were used. The titles and abstracts of all articles were assessed, and irrelevant studies were eliminated. Relevant articles were obtained in full text and evaluated. Hand searches of the reference lists were also performed to identify any relevant articles that were missed with the search strategy.

## 3. Case Series

### 3.1. Case 1: A 60-Year-Old Male Diagnosed with Sigmoid Colon Cancer with Peritoneal Carcinomatosis

The initial pathological evaluation of the biopsy obtained from the primary tumor revealed a poorly differentiated adenocarcinoma. RAS/BRAF testing on tissue using real-time PCR showed a wild-type tumor. The patient received first-line Cetuximab plus FOLFOX (fluorouracil, leucovorin, oxaliplatin), with stable disease at two-month and four-month follow-up. At the time of each imaging evaluation, liquid biopsy was also performed using Idylla© Biocartis, a fully automated real-time-PCR-based molecular diagnostic system and it confirmed the KRAS, NRAS and BRAF wild-type status (Table 1).

The CT scan performed three months later showed progressive disease. Simultaneous liquid biopsy detected a KRAS mutation (G12V) at the time of progression. The patient then started treatment with Bevacizumab and FOLFIRI and had stable disease at the first follow-up. After six months, the patient’s performance status deteriorated, and he was referred to palliative care.

### 3.2. Case 2: A 44-Year-Old Man Diagnosed with Sigmoid Colon Cancer and Synchronous Liver Metastasis

At time of the initial diagnosis, the pathological evaluation of the biopsy specimen from the primary tumor showed a moderately differentiated adenocarcinoma. Genomic testing revealed a KRAS, NRAS, BRAF wild type and a dMMR tumor. RAS/BRAF testing on tissue was performed with real-time PCR, while the MMR status was evaluated by immunohistochemistry. Concomitantly, ctDNA mutational analysis was performed using the Idylla© Biocartis. Surprisingly, the liquid biopsy analysis at baseline detected a KRAS G13D mutation (Table 1).

Given the RAS/BRAF wt status on tissue analysis (which represents the standard of care), the patient received first-line treatment with Cetuximab and FOLFOX. A control MRI was performed three months after systemic treatment initiation and showed a partial response to treatment. Simultaneously, liquid biopsy was repeated, and it revealed this time a KRAS G12D mutation (Table 1). The patient continued the same treatment and the imaging evaluation three months later showed stable disease, while the liquid biopsy detected the same KRAS G12D mutation.

The patient received maintenance treatment with cetuximab and deGramont (fluorouracil, leucovorin). Three months later (9 months from the initial diagnosis), the clinical status of the patients deteriorated rapidly, and the CT scan showed progressive disease. At the time of the imaging evaluation, ctDNA mutational analysis was also repeated and a KRAS G12D mutation was detected (Table 1). The patient was referred to palliative care and died within one month.

### 3.3. Case 3: A 68-Year-Old Female Diagnosed with Sigmoid Colon Cancer with Synchronous Lung and Liver Metastasis

At time of diagnosis, the pathological evaluation of a biopsy specimen from the primary tumor showed a moderately differentiated adenocarcinoma. KRAS, NRAS and BRAF testing on tissue was performed with real-time PCR and revealed a wild-type tumor. At the same time, ctDNA mutational analysis with Idylla© Biocartis confirmed the wild-type status. The patient started treatment with cetuximab and FOLFOX and had partial response at three-month follow up. However, liquid biopsy at the time identified a KRAS mutation (K117N). Patient continued the same treatment and had stable disease at six-month follow up. RAS/BRAF testing using Idylla© Biocartis was repeated at the time of the imaging evaluation and revealed this time a wild-type status. The patient continued maintenance treatment with cetuximab and 5-FU/LV (fluorouracil, leucovorin). Imaging evaluation at nine-month and twelve-months follow-up showed stable disease and the liquid biopsy did not detect any KRAS/NRAS/BRAF mutation. The patient is currently undergoing the same treatment and imagining evaluations and ctDNA mutational analysis will be continued.

## 4. Literature Review

We performed a literature review to assess the degree of agreement between tissue and plasma RAS mutational status in patients with mCRC and the importance of tissue/plasma discordant cases. The main focus was the identification of a KRAS mutation in the plasma of patients diagnosed initially with wild-type mCRC.

Circulating tumor DNA (ctDNA) levels are found to correlate with a number of biological and clinicopathological characteristics, including tumor burden, stage, histotype, apoptotic rate, proximity to blood vessels, and metastatic potential [19,20,21]. A considerable proportion of patients with mCRC display detectable ctDNA in plasma, with mutation rates ranging from 1.9% to 27% [20]. Consequently, the non-invasive identification of emerging KRAS mutations in cell-free DNA (cfDNA) from peripheral blood can facilitate the detection of resistance to anti-EGFR therapy [22].

High concordance between liquid biopsy and tissue biopsy for tumor molecular profiling has been previously demonstrated [23]. A prospective study compared KRAS mutation results obtained from plasma cfDNA testing with those obtained from tumor tissue biopsy and showed that plasma cfDNA analysis exhibited 98% specificity and 92% sensitivity, with a concordance rate of 96% [24]. Numerous studies demonstrated a good concordance between tissue and cfDNA testing of RAS mutations in patients with mCRC [11,25,26,27,28]. Several studies focused on patients with a RAS-positive liquid biopsy but a negative tissue RAS test. A comprehensive presentation of these articles can be found in Table 2. A RAS mutation was identified by liquid biopsy in patients with wild-type mCRC on tissue analysis either at baseline or during treatment with EGFR inhibitors.

The rate of RAS positive cases on liquid biopsy at baseline or following treatment with an anti-EGFR in patients with wild-type mCRC differs across studies, most likely due to the limited number of patients and the different technologies that were used for testing.

The identification of mutations in ctDNA from liquid biopsies requires sensitive techniques. Droplet digital PCR (ddPCR) is a highly sensitive, specific and precise technique that facilitates the identification and quantification of specific DNA alterations in various clinical specimens [37]. Recent studies have shown the efficacy of ddPCR in detecting RAS and BRAF mutations in metastatic colorectal cancer (mCRC). The Poseidon study compared liquid biopsy using ddPCR with standard tissue-based molecular testing for RAS/BRAF genotyping in mCRC patients. The liquid biopsy approach showed faster results (7 days vs. 22 days) and high concordance with standard tissue analysis (83%). This supports the use of liquid biopsy and ddPCR in routine mCRC care, especially for rapid decision-making on first-line therapy [38]. Iris van ’t Erve et al. [39] examined the use of liquid biopsy RAS/BRAF ctDNA analyses in mCRC patients to determine eligibility for anti-EGFR therapy. The results showed a 93% concordance between tissue DNA and liquid biopsy ctDNA mutations, with ddPCR being a part of the diagnostic strategy [39]. When compared to next-generation sequencing (NGS) technology, Demuth et al. [40] concluded in a study measuring KRAS mutations in ctDNA of CRC patients using ddPCR and NGS that both methods have high concordance to tumor genotype (79% and 89%). This study also showed that, in cases with sparse material from mCRC patients, smaller plasma volumes may often be sufficient for KRAS mutation detection by ddPCR [40].

Extended RAS and BRAF mutation analysis using NGS was also explored, establishing the importance of the method in analyzing these mutations. In contrast with PCR-based methods, which only detect known mutations in certain genes, NGS has the benefit of analyzing multiple types of genomic alterations simultaneously. It does not only identify point mutations, but also detects insertions, deletions, gene amplifications and microsatellite instability [41]. In ctDNA sequencing, the sensitivity of detecting mutant allele frequency (MAF) or variant allele frequency (VAF) is pivotal for evaluating the effectiveness of an NGS-based ctDNA profiling assay [42,43]. MAF or VAF measures the proportion of DNA molecules carrying a mutation compared to the total number of molecules with the same allele. These metrics indicate the quantity of ctDNA in relation to cell-free DNAs containing tumor-specific mutant alleles. Thus, a lower detectable MAF signifies greater sensitivity of an NGS assay for ctDNA analysis.

The OncoBEAM platform aims to address the technical challenge of detecting rare DNA molecules by implementing improvements to the PCR technique through a process known as BEAMing [26]. DNA amplification is used to facilitate measurement and enhance the quantity of DNA species. BEAMing enables the detection of uncommon DNA sequences (e.g., those carrying a RAS mutation) with high resolution by dividing the PCR procedure into numerous individual reactions [26]. Bando et al. tested the concordance of the RAS mutational status between plasma ctDNA and tumor tissue in Asian patients diagnosed with mCRC using the OncoBEAM platform. The concordance rate between plasma- and tissue-BEAMing was 86.4% [25]. Similar previous studies showed a concordance of 89.7–93.3%, which indicates that the detection of RAS mutations in the blood through liquid biopsy and BEAMing technology may be a useful replacement to tumor testing [26,28,44].

The Idylla system (Biocartis, Mechelen, Belgium) is a fully automated, real-time PCR-based molecular diagnostics system that can rapidly and without preanalytical DNA extraction identify oncogenic mutations, such as in the KRAS gene, in tissue and plasma samples [45]. Vitiello et al. investigated the use of liquid biopsy for detecting RAS/BRAF mutations in patients with mCRC using the Idylla™ Biocartis platform to evaluate KRAS, NRAS and BRAF mutations in ctDNA. The overall agreement between liquid biopsy results and standard tissue-based NGS analyses was 81.94% [14]. The main characteristics of each method are presented in Table 3.

## 5. Discussion

In this case series, we summarized the clinical courses of three RAS wild-type mCRC patients, who were retested for RAS/BRAF mutations during standard-of-care regimens using liquid biopsy. Repeated biopsies to confirm RAS status are not currently used in routine clinical practice, and thus, changes in RAS mutational status during treatment are not completely understood so far. In this case series, all three patients were initially diagnosed as RAS wild-type mCRC, but a KRAS mutation was detected by liquid biopsy.

Genomic instability plays a critical role in carcinogenesis and cancer resistance to specific treatments, and leads to intra- and intertumoral heterogeneity [46]. Clonal diversity confers a competitive edge to cancer cells by increasing the likelihood of preexisting resistant clones, thereby promoting acquired resistance. Chemotherapies, particularly targeted therapies, often yield partial clinical responses, creating a selective pressure that can induce redistribution among clonal populations.

Due to tumor heterogeneity, clonal redistribution is difficult to capture in a tissue sample that is fixed in time. Moreover, it is not feasible to perform multiple tissue biopsies to detect such clonal redistribution in time. These limitations can be overcome by liquid biopsies performed on serially collected blood samples from patients. Moreover, liquid biopsies represent a better method to obtain a comprehensive molecular profile of the primary and metastatic tumors. On the other hand, the limitations of liquid biopsy include mainly the reliance of results on tumor volume, and the lack of a standard technique for analysis [47].

There are **several possible explanations** for the detection of a KRAS mutation by liquid biopsy in the patients reported in this article. Besides primary resistance mechanisms, the efficacy of anti-EGFR agents in mCRC is limited by the development of molecular alterations producing **acquired resistance** [7,16,48,49]. For instance, it has been demonstrated that a significant percentage of patients with KRAS/NRAS wild-type CRC undergoing treatment with EGFR inhibitors will develop RAS mutations at the time of progression [4]. CRC may contain resistant mutant clones before treatment, and the proportion of these resistant clones increases under treatment [50]. In case 1, we reported a patient with wild-type mCRC who became RAS-mutant at the time of progression. This phenomenon has been confirmed in several reports, although the rate of patients who become RAS-mutant at progression significantly differs among the various studies [10,15,22,30,51,52,53,54].

In case 2, a KRAS mutation was identified by liquid biopsy although the patient had a partial response to treatment with cetuximab and chemotherapy. There are data suggesting that resistant mutations detected at very low variant allele frequency (VAF) represent small subclones of the total tumor burden and do not predict lack of response to anti-EGFR treatment [15]. However, six months after the detection of a KRAS mutation, disease progression was documented by radiological assessment in this patient. Studies have shown that the appearance of resistant KRAS mutant clones could be detected for up to 10 months before radiographic confirmation of disease progression [17].

**Tumor heterogeneity** may also be responsible for the fact that KRAS mutations were found in liquid biopsies but were missed in tumor tissue. In patients with wild-type RAS primary tumors, 10% of the metastatic lesions have RAS mutations [55]. However, current data on the concordance of KRAS mutation status between primary tumors and corresponding metastases are conflicting. Numerous studies investigating inter-tumor heterogeneity (between primary tumors and metastases in the same patient) have demonstrated mutational discordance in up to 30% of cases [24,56,57]. The effectiveness of using KRAS mutation status from the primary tumor alone to predict response to anti-EGFR agents remains a subject of ongoing debate. Thus, additional research is needed to gain a deeper understanding of the interplay between the primary tumor and its distant metastases.

Another possibility is that the KRAS mutations detected by liquid biopsy are false positive alterations due to clonal hematopoiesis, and this may be the case for the third case we reported. Previous studies have shown that genes such as TP53 and KRAS can be mutated in white blood cells [58,59]. KRAS mutations detected in cfDNA were eventually classified as clonal hematopoiesis mutations, and not tumor-derived mutations [59]. Clonal hematopoiesis (CH) arises spontaneously through the accumulation of somatic mutations and the clonal expansion of hematopoietic stem cells during normal aging. The detection of these CH-associated mutations has been characterized as a source of biological stamp in plasma liquid biopsy analyses [58]. Misclassification of cfDNA mutations could lead to ineffective therapeutic strategies. Moreover, interference from CH may contribute to false-positive assessments of cfDNA biomarkers, potentially influencing inappropriate treatment decisions. One method to overcome this challenge could be the simultaneous genotyping of cfDNA and white blood cells DNA [60]. However, the increased expenses linked with paired sequencing could pose a challenge to the widespread introduction of liquid biopsy in clinical settings. Enhanced understanding of the molecular features of clonal hematopoiesis coupled with machine learning could represent the optimal solution for paired sequencing with white blood cells [61].

## 6. Conclusions

Based on available evidence, liquid biopsy represents a novel technique for managing patients with mCRC undergoing treatment with EGFR inhibitors. Several studies have reported a dynamic evolution of RAS/BRAF mutations in mCRC patients treated with first-line EGFR inhibitors and chemotherapy. The identification of RAS mutations in these patients is one of the most frequently identified mechanisms of acquired resistance. However, detecting a KRAS mutation via liquid biopsy can be a false positive due to clonal hematopoiesis. More research is needed to determine whether ctDNA monitoring may help guide therapy options in mCRC patients.

## Figures and Tables

**Table 1:** Patients overview.

Case	Blood Sample Collection Date	KRAS Result on Liquid Biopsy	KRAS Mutation	KRAS Protein	KRAS NC	Cq of KRAS Control	KRAS Gene Cq	Therapy	Date of Imagining Evaluation	Result of Imaging Evaluation
Case 1	2 May 2022	WildType	NA	NA	NA	NA	NA	Cetuximab + FOLFOX	April 2022	Sigmoid colon cancer with peritoneal carcinomatosis (baseline)
Case 1	1 July 2022	WildType	NA	NA	NA	NA	NA	Cetuximab + FOLFOX	June 2022	Stable disease
Case 1	16 September 2022	WildType	NA	NA	NA	NA	NA	Cetuximab + FOLFOX	September 2022	Stable disease
Case 1	22 November 2022	Mutant	G12V	p.Gly12Val	c.3G>T	22.9	34	Cetuximab + FOLFOX	December 2022	Progressive disease
Case 2	22 October 2021	Mutant	G13D	p.Gly13Asp	c.38G>A	21.9	28.7	Cetuximab + FOLFOX	October 2021	Sigmoid colon cancer with liver metastases (baseline)
Case 2	29 December 2021	Mutant	G12D	pGly12Asp	c.35G>A	23	32	Cetuximab + FOLFOX	January 2022	Partial response
Case 2	23 February 2022	Mutant	G12D	pGly12Asp	c.35G>A	23	32.1	Cetuximab + FOLFOX	March 2022	Stable disease
Case 2	29 June 2022	Mutant	G12D	pGly12Asp	c.35G>A	23	30.4	Cetuximab + FOLFOX	June 2022	Progressive disease
Case 3	15 February 2023	WildType	NA	NA	NA	NA	NA	Panitumumab + FOLFOX	20 January 2023	Sigmoid colon cancer with liver and lung metastasis (baseline)
Case 3	24 April 2023	Mutant	K117N	p.Lys117Asn	c.351A>C; c.351A>T	21.3	33.9	Panitumumab + FOLFOX	March 2023	Partial response
Case 3	20 June 2023	WildType	NA	NA	NA	NA	NA	Panitumumab + FOLFOX	June 2023	Stable disease
Case 3	2 October 2023	WildType	NA	NA	NA	NA	NA	5-FU/LV+ FOLFOX	October 2023	Stable disease
Case 4	5 January 2024	WildType	NA	NA	NA	NA	NA	5-FU/LV + FOLFOX	December 2023	Stable disease

NA, not applicable; FOLFOX, 5-FU, leucovorin, oxaliplatin; Cq, cycle of quantification value.

**Table 2:** Published studies of dynamic molecular monitoring by liquid biopsy (ctDNA) in patients with RAS wild-type metastatic colorectal cancer.

Study	No. of Patients RAS wt at the Baseline	ctDNA Analysis Method	No. of Patients with RAS Mutations Detected by Liquid Biopsy
Diaz et al. 2012 [16]	24	BEAMing	9 (38%)
Morelli et al., 2015 [29]	62	BEAMing	27 (43%)
Vidal et al., 2017 [15]	18	BEAMing	7 (39%)
Toledo et al., 2017 [30]	23	BEAMing	2 (9%)
Normanno et al., 2017 [31]	92	BEAMing	33 (36%)
Pietrantonio et al., 2017 [32]	11	ddPCR	4 (36%)
Strickler et al., 2018 [9]	42	NGS	26 (62%)
Kim et al., 2018 [10]	164	NGS	53 (32.3%)
Takayama et al., 2018 [33]	25	ddPCR	9 (36%)
Vitiello et al., 2019 [14]	30	RT-qPCR	10 (30%)
Yamada et al., 2020 [34]	19	ddPCR	16 (84%)
Lim et al., 2021 [35]	93	NGS	7 (7.5%)
Rachiglio et al., 2022 [36]	37	Idylla	6 (16%)

**Table 3:** Overview of technologies used for detection of KRAS mutation in ctDNA.

Feature	ddPCR	Idylla	BEAMing	NGS
Sensitivity and Specificity	High; excellent for low-frequency mutations	Good; slightly lower for very low-frequency mutations	Very high; comparable to ddPCR	High; dependent on sequencing depth
Turnaround Time	Relatively fast	Very fast (within hours)	Slower due to complex procedure	Slowest (days to weeks)
Ease of Use	Requires technical expertise	Highly automated and user-friendly	Technically demanding, specialized equipment needed	Requires specialized expertise and complex data analysis
Cost	Moderate; less expensive than NGS	Moderate; cost-effective for single mutation testing	High	High; especially for comprehensive sequencing
Application	Quantifying mutation frequency, detecting minimal residual disease	Rapid, routine testing, single mutation analysis	Highly sensitive detection of low-frequency mutations; less practical for routine use	Comprehensive mutation profiling, identification of novel mutations, multiplex capability
Suitability for Clinical Use	Suitable for specific mutation quantification	Ideal for rapid decision-making in clinical settings	More suited for research settings	Comprehensive analysis, suited for in-depth research and complex cases

## Supplementary Materials

The following supporting information can be downloaded at: https://www.mdpi.com/article/10.3390/jpm14070750/s1, Liquid biopsy analysis using Idylla fully automated, real-time PCR based molecular testing system.

## References

[1] International Agency for Research on Cancer Global Cancer Observatory (GLOBOCAN). https://gco.iarc.who.int/media/globocan/factsheets/populations/900-world-fact-sheet.pdf.

[2] Cervantes A., Adam R., Roselló S., Arnold D., Normanno N., Taïeb J., Seligmann J., De Baere T., Osterlund P., Yoshino T. (2023). Metastatic colorectal cancer: ESMO Clinical Practice Guideline for diagnosis, treatment and follow-up. Ann. Oncol..

[3] Mendelsohn J., Prewett M., Rockwell P., Goldstein N.I. (2015). CCR 20th anniversary commentary: A chimeric antibody, C225, inhibits EGFR activation and tumor growth. Clin. Cancer Res..

[4] Yarom N., Jonker D.J. (2011). The role of the epidermal growth factor receptor in the mechanism and treatment of colorectal cancer. Discov. Med..

[5] Guerrera L.P., Napolitano S., De Falco V., Giunta E.F., Vitiello P.P., Gravina A.G., Suarato G., Perrone A., Napolitano R., Martinelli E. (2021). Multiple Acquired Mutations Captured by Liquid Biopsy in the EGFR Addicted Metastatic Colorectal Cancer. Clin. Color. Cancer.

[6] Siravegna G., Marsoni S., Siena S., Bardelli A. (2017). Integrating liquid biopsies into the management of cancer. Nat. Rev. Clin. Oncol..

[7] Patelli G., Mauri G., Tosi F., Amatu A., Bencardino K., Bonazzina E., Pizzutilo E.G., Villa F., Calvanese G., Agostara A.G. (2023). Circulating tumor DNA to drive treatment in metastatic colorectal cancer. Clin. Cancer Res..

[8] Gerlinger M., Rowan A.J., Horswell S., Larkin J., Endesfelder D., Gronroos E., Martinez P., Matthews N., Stewart A., Tarpey P. (2012). Intratumor heterogeneity and branched evolution revealed by multiregion sequencing. N. Engl. J. Med..

[9] Strickler J.H., Loree J.M., Ahronian L.G., Parikh A.R., Niedzwiecki D., Pereira A.A.L., McKinney M., Korn W.M., Atreya C.E., Banks K.C. (2018). Genomic landscape of cell-free DNA in patients with colorectal cancer. Cancer Discov..

[10] Kim T.W., Peeters M., Thomas A., Gibbs P., Hool K., Zhang J., Ang A.L., Bach B.A., Price T. (2018). Impact of emergent circulating tumor DNA RAS mutation in panitumumab-treated chemoresistant metastatic colorectal cancer. Clin. Cancer Res..

[11] Bachet J., Bouché O., Taieb J., Dubreuil O., Garcia M., Meurisse A., Normand C., Gornet J., Artru P., Louafi S. (2018). RAS mutation analysis in circulating tumor DNA from patients with metastatic colorectal cancer: The AGEO RASANC prospective multicenter study. Ann. Oncol..

[12] Furuki H., Yamada T., Takahashi G., Iwai T., Koizumi M., Shinji S., Yokoyama Y., Takeda K., Taniai N., Uchida E. (2018). Evaluation of liquid biopsies for detection of emerging mutated genes in metastatic colorectal cancer. Eur. J. Surg. Oncol..

[13] Lastraioli E., Bettiol A., Iorio J., Limatola E., Checcacci D., Parisi E., Bianchi C., Arcangeli A., Iannopollo M., Di Costanzo F. (2023). Evaluation of RAS Mutational Status in Liquid Biopsy to Monitor Disease Progression in Metastatic Colorectal Cancer Patients. Cells.

[14] Vitiello P.P., De Falco V., Giunta E.F., Ciardiello D., Cardone C., Vitale P., Zanaletti N., Borrelli C., Poliero L., Terminiello M. (2019). Clinical practice use of liquid biopsy to identify RAS/BRAF mutations in patients with metastatic colorectal cancer (mCRC): A single institution experience. Cancers.

[15] Vidal J., Muinelo L., Dalmases A., Jones F., Edelstein D., Iglesias M., Orrillo M., Abalo A., Rodríguez C., Brozos E. (2017). Plasma ctDNA RAS mutation analysis for the diagnosis and treatment monitoring of metastatic colorectal cancer patients. Ann. Oncol..

[16] Diaz Jr L.A., Williams R.T., Wu J., Kinde I., Hecht J.R., Berlin J., Allen B., Bozic I., Reiter J.G., Nowak M.A. (2012). The molecular evolution of acquired resistance to targeted EGFR blockade in colorectal cancers. Nature.

[17] Misale S., Yaeger R., Hobor S., Scala E., Janakiraman M., Liska D., Valtorta E., Schiavo R., Buscarino M., Siravegna G. (2012). Emergence of KRAS mutations and acquired resistance to anti-EGFR therapy in colorectal cancer. Nature.

[18] Chibaudel B. (2018). Extended RAS mutational status analysis in circulating tumor DNA from patients with advanced colorectal cancer in daily clinical practice. The Franco-British Institute Experience and Recommendations. Biomed. J. Sci. Tech. Res..

[19] Diehl F., Schmidt K., Durkee K.H., Moore K.J., Goodman S.N., Shuber A.P., Kinzler K.W., Vogelstein B.J.G. (2008). Analysis of mutations in DNA isolated from plasma and stool of colorectal cancer patients. Gastroenterology.

[20] Bettegowda C., Sausen M., Leary R.J., Kinde I., Wang Y., Agrawal N., Bartlett B.R., Wang H., Luber B., Alani R.M. (2014). Detection of circulating tumor DNA in early-and late-stage human malignancies. Sci. Transl. Med..

[21] El Messaoudi S., Mouliere F., Du Manoir S., Bascoul-Mollevi C., Gillet B., Nouaille M., Fiess C., Crapez E., Bibeau F., Theillet C.J.C.C.R. (2016). Circulating DNA as a strong multimarker prognostic tool for metastatic colorectal cancer patient management care. Clin. Cancer Res..

[22] Siravegna G., Mussolin B., Buscarino M., Corti G., Cassingena A., Crisafulli G., Ponzetti A., Cremolini C., Amatu A., Lauricella C. (2015). Clonal evolution and resistance to EGFR blockade in the blood of colorectal cancer patients. Nat. Med..

[23] Peeters M., Price T., Boedigheimer M., Kim T.W., Ruff P., Gibbs P., Thomas A., Demonty G., Hool K., Ang A. (2019). Evaluation of emergent mutations in circulating cell-free DNA and clinical outcomes in patients with metastatic colorectal cancer treated with panitumumab in the ASPECCT study. Clin. Cancer Res..

[24] Thierry A.R., Mouliere F., El Messaoudi S., Mollevi C., Lopez-Crapez E., Rolet F., Gillet B., Gongora C., Dechelotte P., Robert B. (2014). Clinical validation of the detection of KRAS and BRAF mutations from circulating tumor DNA. Nat. Med..

[25] Bando H., Kagawa Y., Kato T., Akagi K., Denda T., Nishina T., Komatsu Y., Oki E., Kudo T., Kumamoto H. (2019). A multicentre, prospective study of plasma circulating tumour DNA test for detecting RAS mutation in patients with metastatic colorectal cancer. Br. J. Cancer.

[26] García-Foncillas J., Tabernero J., Élez E., Aranda E., Benavides M., Camps C., Jantus-Lewintre E., López R., Muinelo-Romay L., Montagut C. (2018). Prospective multicenter real-world RAS mutation comparison between OncoBEAM-based liquid biopsy and tissue analysis in metastatic colorectal cancer. Br. J. Cancer.

[27] Germetaki T., Nicholls C., Adams R.A., Braun M., Rogan J., Moghadam S., Lenfert E., Lukas A., Edelstein D.L., Jones F.S. (2020). Blood-based RAS mutation testing: Concordance with tissue-based RAS testing and mutational changes on progression. Future Oncol..

[28] Grasselli J., Elez E., Caratù G., Matito J., Santos C., Macarulla T., Vidal J., Garcia M., Viéitez J., Páez D. (2017). Concordance of blood-and tumor-based detection of RAS mutations to guide anti-EGFR therapy in metastatic colorectal cancer. Ann. Oncol..

[29] Morelli M.P., Overman M.J., Dasari A., Kazmi S.M.A., Mazard T., Vilar E., Morris V.K., Lee M.S., Herron D., Eng C. (2015). Characterizing the patterns of clonal selection in circulating tumor DNA from patients with colorectal cancer refractory to anti-EGFR treatment. Ann. Oncol..

[30] Toledo R.A., Cubillo A., Vega E., Garralda E., Alvarez R., de la Varga L.U., Pascual J.R., Sánchez G., Sarno F., Prieto S.H. (2017). Clinical validation of prospective liquid biopsy monitoring in patients with wild-type RAS metastatic colorectal cancer treated with FOLFIRI-cetuximab. Oncotarget.

[31] Normanno N., Esposito Abate R., Lambiase M., Forgione L., Cardone C., Iannaccone A., Sacco A., Rachiglio A.M., Martinelli E., Rizzi D. (2018). RAS testing of liquid biopsy correlates with the outcome of metastatic colorectal cancer patients treated with first-line FOLFIRI plus cetuximab in the CAPRI-GOIM trial. Ann. Oncol..

[32] Pietrantonio F., Vernieri C., Siravegna G., Mennitto A., Berenato R., Perrone F., Gloghini A., Tamborini E., Lonardi S., Morano F. (2017). Heterogeneity of acquired resistance to anti-EGFR monoclonal antibodies in patients with metastatic colorectal cancer. Clin. Cancer Res..

[33] Takayama Y., Suzuki K., Muto Y., Ichida K., Fukui T., Kakizawa N., Ishikawa H., Watanabe F., Hasegawa F., Saito M. (2018). Monitoring circulating tumor DNA revealed dynamic changes in KRAS status in patients with metastatic colorectal cancer. Oncotarget.

[34] Yamada T., Matsuda A., Takahashi G., Iwai T., Takeda K., Ueda K., Kuriyama S., Koizumi M., Shinji S., Yokoyama Y. (2020). Emerging RAS, BRAF, and EGFR mutations in cell-free DNA of metastatic colorectal patients are associated with both primary and secondary resistance to first-line anti-EGFR therapy. Int. J. Clin. Oncol..

[35] Lim Y., Kim S., Kang J.-K., Kim H.-P., Jang H., Han H., Kim H., Kim M.J., Lee K.-H., Ryoo S.-B. (2021). Circulating tumor DNA sequencing in colorectal cancer patients treated with first-line chemotherapy with anti-EGFR. Sci. Rep..

[36] Rachiglio A.M., Forgione L., Pasquale R., Barone C.A., Maiello E., Antonuzzo L., Cassata A., Tonini G., Bordonaro R., Rosati G. (2022). Dynamics of RAS/BRAF Mutations in cfDNA from Metastatic Colorectal Carcinoma Patients Treated with Polychemotherapy and Anti-EGFR Monoclonal Antibodies. Cancers.

[37] Olmedillas-López S., García-Arranz M., García-Olmo D. (2017). Current and emerging applications of droplet digital PCR in oncology. Mol. Diagn. Ther..

[38] Procaccio L., Bergamo F., Daniel F., Rasola C., Munari G., Biason P., Crucitta S., Barsotti G., Zanella G., Angerilli V. (2021). A real-world application of liquid biopsy in metastatic colorectal cancer: The Poseidon study. Cancers.

[39] van’t Erve I., Greuter M.J., Bolhuis K., Vessies D.C., Leal A., Vink G.R., van den Broek D., Velculescu V.E., Punt C.J., Meijer G.A. (2020). Diagnostic strategies toward clinical implementation of liquid biopsy RAS/BRAF circulating tumor DNA analyses in patients with metastatic colorectal cancer. J. Mol. Diagn..

[40] Demuth C., Spindler K.-L.G., Johansen J.S., Pallisgaard N., Nielsen D., Hogdall E., Vittrup B., Sorensen B.S. (2018). Measuring KRAS mutations in circulating tumor DNA by droplet digital PCR and next-generation sequencing. Transl. Oncol..

[41] Jing C., Mao X., Wang Z., Sun K., Ma R., Wu J., Cao H.J.M.m.r. (2018). Next-generation sequencing-based detection of EGFR, KRAS, BRAF, NRAS, PIK3CA, Her-2 and TP53 mutations in patients with non-small cell lung cancer. Mol. Med. Rep..

[42] Stewart C.M., Kothari P.D., Mouliere F., Mair R., Somnay S., Benayed R., Zehir A., Weigelt B., Dawson S.J., Arcila M.E. (2018). The value of cell-free DNA for molecular pathology. J. Pathol..

[43] Bos M.K., Nasserinejad K., Jansen M.P., Steendam C.M., Angus L., Atmodimedjo P.N., de Jonge E., Dinjens W.N., van Schaik R.H., Del Re M.J.M.O. (2021). Comparison of variant allele frequency and number of mutant molecules as units of measurement for circulating tumor DNA. Mol. Oncol..

[44] Schmiegel W., Scott R.J., Dooley S., Lewis W., Meldrum C.J., Pockney P., Draganic B., Smith S., Hewitt C., Philimore H. (2017). Blood-based detection of RAS mutations to guide anti-EGFR therapy in colorectal cancer patients: Concordance of results from circulating tumor DNA and tissue-based RAS testing. Mol. Oncol..

[45] Uguen A., Troncone G. (2018). A review on the Idylla platform: Towards the assessment of actionable genomic alterations in one day. J. Clin. Pathol..

[46] Klein-Scory S., Maslova M., Pohl M., Eilert-Micus C., Schroers R., Schmiegel W., Baraniskin A. (2018). Significance of Liquid Biopsy for Monitoring and Therapy Decision of Colorectal Cancer. Transl. Oncol..

[47] Merker J.D., Oxnard G.R., Compton C., Diehn M., Hurley P., Lazar A.J., Lindeman N., Lockwood C.M., Rai A.J., Schilsky R.L. (2018). Circulating tumor DNA analysis in patients with cancer: American Society of Clinical Oncology and College of American Pathologists joint review. Arch. Pathol. Lab. Med..

[48] Arena S., Bellosillo B., Siravegna G., Martínez A., Canadas I., Lazzari L., Ferruz N., Russo M., Misale S., González I. (2015). Emergence of multiple EGFR extracellular mutations during cetuximab treatment in colorectal cancer. Clin. Cancer Res..

[49] Bertotti A., Sassi F. (2015). Molecular pathways: Sensitivity and resistance to anti-EGFR antibodies. Clin. Cancer Res..

[50] Osumi H., Shinozaki E., Yamaguchi K., Zembutsu H. (2019). Clinical utility of circulating tumor DNA for colorectal cancer. Cancer Sci..

[51] Van Emburgh B.O., Arena S., Siravegna G., Lazzari L., Crisafulli G., Corti G., Mussolin B., Baldi F., Buscarino M., Bartolini A. (2016). Acquired RAS or EGFR mutations and duration of response to EGFR blockade in colorectal cancer. Nat. Commun..

[52] Thomsen C.B., Andersen R.F., Lindebjerg J., Hansen T.F., Jensen L.H., Jakobsen A. (2019). Plasma dynamics of RAS/RAF mutations in patients with metastatic colorectal cancer receiving chemotherapy and anti-EGFR treatment. Clin. Color. Cancer.

[53] Siena S., Sartore-Bianchi A., García-Carbonero R., Karthaus M., Smith D., Tabernero J., Van Cutsem E., Guan X., Boedigheimer M., Ang A. (2018). Dynamic molecular analysis and clinical correlates of tumor evolution within a phase II trial of panitumumab-based therapy in metastatic colorectal cancer. Ann. Oncol..

[54] Maurel J., Alonso V., Escudero P., Fernández-Martos C., Salud A., Méndez M., Gallego J., Rodriguez J.R., Martín-Richard M., Fernández-Plana J. (2019). Clinical impact of circulating tumor RAS and BRAF Mutation dynamics in patients with metastatic colorectal cancer treated with first-line chemotherapy plus anti–epidermal growth factor receptor therapy. JCO Precis. Oncol..

[55] Mao C., Wu X.-Y., Yang Z.-Y., Threapleton D.E., Yuan J.-Q., Yu Y.-Y., Tang J.-L. (2015). Concordant analysis of KRAS, BRAF, PIK3CA mutations and PTEN expression between primary colorectal cancer and matched metastases. Sci. Rep..

[56] Knijn N., Mekenkamp L., Klomp M., Vink-Börger M., Tol J., Teerenstra S., Meijer J., Tebar M., Riemersma S., Van Krieken J. (2011). KRAS mutation analysis: A comparison between primary tumours and matched liver metastases in 305 colorectal cancer patients. Br. J. Cancer.

[57] Kim M.-J., Lee H.S., Kim J.H., Kim Y.J., Kwon J.H., Lee J.-O., Bang S.-M., Park K.U., Kim D.-W., Kang S.-B. (2012). Different metastatic pattern according to the KRAS mutational status and site-specific discordance of KRAS status in patients with colorectal cancer. BMC Cancer.

[58] Chan H.T., Chin Y.M., Nakamura Y., Low S.-K. (2020). Clonal hematopoiesis in liquid biopsy: From biological noise to valuable clinical implications. Cancers.

[59] Hu Y., Ulrich B.C., Supplee J., Kuang Y., Lizotte P.H., Feeney N.B., Guibert N.M., Awad M.M., Wong K.-K., Jänne P.A. (2018). False-positive plasma genotyping due to clonal hematopoiesis. Clin. Cancer Res..

[60] Croitoru V.M., Cazacu I.M., Popescu I., Paul D., Dima S.O., Croitoru A.E., Tanase A.D. (2022). Clonal hematopoiesis and liquid biopsy in gastrointestinal cancers. Front. Med..

[61] Chabon J.J., Hamilton E.G., Kurtz D.M., Esfahani M.S., Moding E.J., Stehr H., Schroers-Martin J., Nabet B.Y., Chen B., Chaudhuri A.A.J.N. (2020). Integrating genomic features for non-invasive early lung cancer detection. Nature.

