# Supplementary material for: Dynamics of RAS Mutations in Liquid Biopsies in Metastatic Colorectal Cancer Patients—Case Series and Literature Review
Source: J Pers Med. 2024 Jul 15;14(7):750. doi: 10.3390/jpm14070750 (PMC11278408; doi:10.3390/jpm14070750)
Supplement: Supplementary file 1 [file jpm-14-00750-s001.zip › jpm-3006960-supplementary.pdf]

Liquid biopsy analysis using Idylla fully automated, real-time PCR based molecular testing system.

1. Objective

Liquid biopsy analysis using IdyllaTM Platform (Biocartis) – a fully automated, real-time PCR based molecular testing system, to establish the RAS status (mutant or wild type) in metastatic colorectal cancer subjects using ctKRAS and ctNRAS-BRAF cartridges with the Idylla system from liquid biopsy samples collected in a prospective study.

2. Materials and Reagents:

A) Liquid Biopsy Collection:

- Blood collection tubes (K2EDTA tubes or Streck Cell-Free DNA BCT) – *prelabeled with barcode, subject ID and sample no.*
- Collection supplies (e.g., 21G needles & holders, tourniquets, alcohol swabs)

B) Reagents:

- ctKRAS cartridge (Catalog# A0080/6, Biocartis) - 21 clinically relevant mutations:

KRAS mutation detection	
Codon 12 (exon 2)	G12C (c.34G>T)
	G12R (c.34G>C)
	G12S (c.34G>A)
	G12A (c.35G>C)
	G12D (c.35G>A)
	G12V (c.35G>T)
Codon 13 (exon 2)	G13D (c.38G>A)
Codon 59 (exon 3)	A59E (c.176C>A)
	A59G (c.176C>G)
	A59T (c.175G>A)
Codon 61 (exon 3)	Q61K (c.181C>A; c.180_181delinsAA)
	Q61L (c.182A>T)
	Q61R (c.182A>G)
	Q61H (c.183A>C; c.183A>T)
Codon 117 (exon 4)	K117N (c.351A>C; c.351A>T)
Codon 146 (exon 4)	A146P (c.436G>C)
	A146T (c.436G>A)
	A146V (c.437C>T)

Figure 1 - ctKRAS mutation detection.

- ctNRAS-BRAF cartridge (Catalog# A0030/6, Biocartis) - 23 clinically relevant mutations:

NRAS mutation detection	
Codon 12 (exon 2)	G12C (c.34G>T)

	G12S (c.34G>A)
	G12D (c.35G>A)
	G12A (c.35G>C)
	G12V (c.35G>T)
Codon 13 (exon 2)	G13D (c.38G>A)
	G13V (c.38G>T)
	G13R (c.37G>C)
Codon 59 (exon 3)	A59T (c.175G>A)
Codon 61 (exon 3)	Q61K (c.181C>A)
	Q61R (c.182A>G)
	Q61L (c.182A>T)
	Q61H (c.183A>C; c.183A>T)
Codon 117 (exon 4)	K117N (c.351G>C; c.351G>T)
Codon 146 (exon 4)	A146T (c.436G>A)
	A146V (c.437C>T)
**BRAF mutation detection**	
Codon 600	BRAF V600E (c.1799T>A; c.1799_1800delinsAA)
	BRAF V600D (c.1799_1800delinsAC)
	BRAF V600K (c.1798_1799delinsAA)
	BRAF V600R (c.1798_1799delinsAG)

Figure 2 - ctNRAS-BRAF mutation detection

C) Equipment:

- IdyllaTM Platform (including the analysis module)
- Centrifuge (for plasma separation)
- Eppendorf Safe-Lock Tubes PCR clean (DNase-free, RNase-free and PCR inhibitors free; Catalog No. 022363344, Eppendorf)
- Pipet-Lite LTS Pipette L-1000XLS+ (Catalog Nr. 17014382, Mettler Toledo) with Pipette Tips RT LTS 1000µL FL 768A/8 (Catalog Nr. 30389213, Mettler Toledo)
- Personal protective equipment (PPE)

3. Prerequisites

A) Liquid Biopsy Collection Procedure

Serial whole blood samples (from ~ 2 to 2 months intervals) will be prospectively collected from enrolled mCRC subjects who consent and who fulfil the inclusion/exclusion criteria of study protocol. For each subject there will be one (1) vacutainer collected per visit.

Blood draw (at least 7ml) will be performed using standard procedure of peripheral vein blood draw, using Streck Cell-Free DNA BCT CE 10ml (Streck Catalog Nr. 218997) or BD K2EDTA Vacutainer® 10ml (BD Catalog Nr. 367525).

The collected vacutainer will be placed upright in a rack at room temperature until pre-analytical processing.

<u>Note:</u>

- *If using **<u>K2EDTA tubes</u>**, the blood must be centrifuged within **<u>4h after collection</u>**.*
- *If using **<u>Streck Cell-Free DNA BCT tubes</u>**, the blood must be centrifuged within **<u>3 days of collection</u>**.*

B) Pre-analytical processing

A centrifuge with swing-out rotor capable of providing a relative centrifuge force between 1600-6000g and buckets to accommodate for 10ml vacutainers and 2 ml Eppendorf tubes will be used to double spin the biospecimen.

Pre-analytical processing of samples will be carried out by qualified personnel as per Biocartis Idylla RT-PCR platform instructions of pre-analytical sample processing for ctDNA analysis:

1. Collected BD K2EDTA or Streck Cell-Free DNA BCT CE vacutainers will be placed in a centrifuge with a swing-out rotor and centrifuged for 10 minutes at 1600g (RCF) at room temperature having the centrifuge brake OFF.
2. Immediately after centrifugation, plasma will be carefully collected, avoiding the buffy coat layer by salting 0.5 cm plasma above it, and 1.2 ml aliquoted in 2 ml Eppendorf Safe-Lock Tubes of all available plasma.
3. Resulting Eppendorf Safe-Lock Tubes will be then centrifuged for 10 minutes at 6000g (RCF) or 60 seconds at 15000-20000g (RCF) at room temperature (medium brake setting).
4. Plasma will be carefully collected after the second centrifugation, avoiding accidental suction of platelets by leaving 100µl above the platelet layer. All resulting plasma will be aliquoted: 1.1ml in 2ml Eppendorf Safe-Lock Tubes.
5. The resulting Eppendorf Safe-Lock Tubes will be immediately tested using the Biocartis Idylla RT-PCR platform or stored for future testing in the freezer at -80°C.

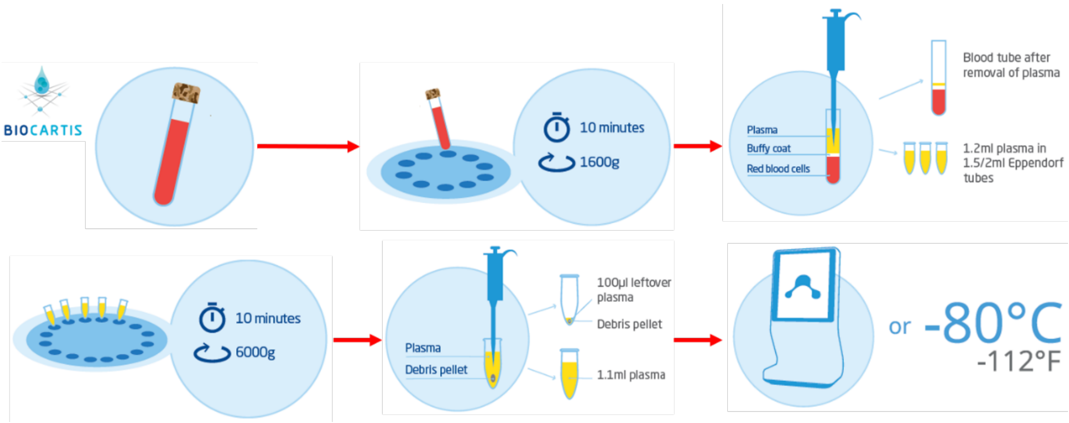

Figure 3 - Pre-analytical processing steps

4. Sample analysis using IdyllaTM Platform (Biocartis)

The separated plasma will be tested using the IdyllaTM Platform using specific cartridges (Idylla™ ctKRAS Mutation Test and Idylla™ ctNRAS-BRAF Mutation Test) to determine the mutational status of KRAS, NRAS and BRAF of the ctDNA in patient sample.

The detection limit of the Idylla RT-PCR platform for cfDNA is specified to be about 10,000 copies of WT DNA (about 30 ng) per milliliter. When the test is completed, the platform displays an automated report that includes: validity of the run (in case no DNA is detected, the test is considered invalid), presence or absence of mutation(s), type of mutation(s) detected, Cq of the total KRAS/NRAS/BRAF, Cq of the specific mutation(s) detected.

The results of the genetic testing will be analyzed in correlation with the relevant medical information collected through the patient's CRF.

Steps followed on Idylla RT-PCR platform to perform the testing of a sample:

a) The machine must be placed on a dedicated and stable table.
b) Start the Console from the ON/OFF button located at the top right side of the Console.
c) Start the instrument from the ON/OFF button located on its back. The device starts with alternative red and white lights around the sample drawer. The initialization takes about a minute. When the initialization is finished, the white light under the drawer shows that the tool is turned on and idle.
d) To connect to Console, take the following steps:
- On the Main screen, press LOGIN.
- Enter NAME & PASSWORD.
- Press OK.
e) To create a manual TEST request, follow the next steps:
- Press NEW TEST.
- Scan the barcode of the sample (when the light from the console scanner is turned on) or you can manually enter the sample ID. The sample ID is automatically displayed with the barcode/sample name.
- Scan the barcode in the upper part of the Test Cartridge.
- The details of the Test are displayed in the information area of the cartridge.
- The system automatically feeds the Test Type and Sample Type.
- Optionally, enter a COMMENT as an annotation when requesting TEST and RESULTS.
- Press CONFIRM to complete the TEST request.

When the TEST request is completed, the system reserves an instrument. The white light ring of the preferred instrument is starting to flash. If no instrument is available at the time, the TEST request is stored in a processing que.

f) When the sample is processed according to the protocol, it is inserted into the cartridge and a TEST request is created, and then the cartridge is loaded into the Instrument.
- Press the open/closed button on the Instrument drawer.
- Place the cartridge in the Instrument drawer.
- Press the open button again on the instrument drawer.

You can monitor the progress of the Test on the STATUS screen. A progress bar appears that shows the remaining time of the Test in hours and minutes. To view the Test's progress in more detail, click on the STATUS on the instrument screen. The TEST IN PROGRESS screen opens. As soon as the Test is completed, the progress bar is replaced by the RESULTS state. Press on the status bar to get more details and to view the Test result (a generated document can be downloaded).

g) When a Test is completed, reopen the Instrument drawer, and dispose the processed Cartridge as biological risk material.
h) After job is finished, press the Log Off button and close the console. To close the instrument, press the ON/OFF button in position 0.
